# The association between a conditional cash transfer programme and malaria incidence: a longitudinal ecological study in the Brazilian Amazon between 2004 and 2015

**DOI:** 10.1186/s12889-021-11255-0

**Published:** 2021-06-29

**Authors:** Layana Costa Alves, Mauro Niskier Sanchez, Thomas Hone, Luiz Felipe Pinto, Joilda Silva Nery, Pedro Luiz Tauil, Maurício Lima Barreto, Gerson Oliveira Penna

**Affiliations:** 1 Fiocruz School of Government, Oswaldo Cruz Foundation, EFG/FIOCRUZ, Avenida L3 Norte, s/n, Campus Universitário Darcy Ribeiro, Gleba A, Brasília/DF, CEP: 70.904-130 Brazil; 2 Institute of Collective Health, Federal University of Bahia, UFBA, Rua Basílio da Gama, s/n, Campus Universitário Canela, Salvador/BA, CEP: 40.110-040 Brazil; 3 Department of Collective Health, University of Brasília, UNB, Campus Universitário Darcy Ribeiro, s/n, Asa Norte, Brasília/DF, CEP: 70910-900 Brazil; 4 Tropical Medicine Centre, University of Brasília, UNB, Campus Universitário Darcy Ribeiro, s/n, Asa Norte, Brasília/DF, CEP: 70.904.970 Brazil; 5 Public Health Policy Evaluation Unit, Imperial College, Imperial College London, Charing Cross Hospital, London, W6 8RP UK; 6 Department of Medicine in Primary Health Care, School of Medicine, Federal University of Rio de Janeiro, UFRJ, Rua Laura de Araújo, 36 – 2 andar. Cidade Nova, Rio de Janeiro/RJ, CEP: 20211-170 Brazil; 7 Postdoctoral Fellow in the Institute of Hygiene and Tropical Medicine at Nova Medical School, R. da Junqueira 100, 1349-008 Lisbon, Portugal; 8 Department of Collective Health, Federal University of Vale do São Francisco, UNIVASF, Rua da Aurora, s/n, General Dutra, Paulo Afonso/BA, CEP: 48607-190 Brazil; 9 Center for Data and Knowledge Integration for Health, CIDACS, Oswaldo Cruz Foundation, FIOCRUZ, Rua Mundo, 121, Trobogy, Salvador/BA, CEP: 41745-715 Brazil

**Keywords:** Conditional cash transfer, Epidemiology, Malaria, Prevention & control, Social determinants of health, Vector borne disease

## Abstract

**Background:**

Malaria causes 400 thousand deaths worldwide annually. In 2018, 25% (187,693) of the total malaria cases in the Americas were in Brazil, with nearly all (99%) Brazilian cases in the Amazon region. The Bolsa Família Programme (BFP) is a conditional cash transfer (CCT) programme launched in 2003 to reduce poverty and has led to improvements in health outcomes. CCT programmes may reduce the burden of malaria by alleviating poverty and by promoting access to healthcare, however this relationship is underexplored. This study investigated the association between BFP coverage and malaria incidence in Brazil.

**Methods:**

A longitudinal panel study was conducted of 807 municipalities in the Brazilian Amazon between 2004 and 2015. Negative binomial regression models adjusted for demographic and socioeconomic covariates and time trends were employed with fixed effects specifications.

**Results:**

A one percentage point increase in municipal BFP coverage was associated with a 0.3% decrease in the incidence of malaria (RR = 0.997; 95% CI = 0.994–0.998). The average municipal BFP coverage increased 24 percentage points over the period 2004–2015 corresponding to be a reduction of 7.2% in the malaria incidence.

**Conclusions:**

Higher coverage of the BFP was associated with a reduction in the incidence of malaria. CCT programmes should be encouraged in endemic regions for malaria in order to mitigate the impact of disease and poverty itself in these settings.

**Supplementary Information:**

The online version contains supplementary material available at 10.1186/s12889-021-11255-0.

## Background

Malaria is a major health burden that occurs mainly in the tropical regions of Africa, Asia and South America – the most impoverished regions of the planet. Approximately half of the world population is at risk of contracting malaria. In 2018, there were 228 million cases and 405,000 deaths due to malaria worldwide. The majority (67%) of deaths occurred among children under 5 years old [1].

Of all malaria cases in the Americas in 2018, 25% were in Brazil [1]. The endemic area for malaria coincides with rural zones in the Brazilian Amazon, the poorest region of the country [2, 3]. In 2015, Brazil registered 138,199 new autochthonous cases – a case contracted locally with no evidence of importation and no direct link to transmission from an imported case. Ninety-nine per cent of these originated in nine states in the North (Acre, Amapá, Amazonas, Pará, Rondônia, Roraima and Tocantins), Central-West (Mato Grosso) and Northeast (Maranhão) regions of the country. Moreover, 89% of these infections were due to *Plasmodium vivax*, while the rest were caused mainly by *P. falciparum* or mixed infections [3, 4].

The risk of contracting malaria is not homogeneous across the Amazon region. In 2015, from a total of 807 Brazilian municipalities in the region, 29 were considered at high risk — an Annual Parasite Index (API; confirmed annual malaria cases per thousand inhabitants) greater than 50 cases per 1000 inhabitants. Forty municipalities were classified as medium risk (an API between 50 and 10) and 738 as low risk (an API less than 10). Migration, mineral and vegetal exploration, development projects and disorganized settlement are important factors which increase the risk of malaria infection [3, 5–10].

The Bolsa Família Programme (BFP) is a conditional cash transfer (CCT) programme launched in 2003 in Brazil to fight poverty and social inequalities. The programme has three main pillars: a) income support: monthly cash transfers for beneficiary families in order to promote immediate poverty alleviation; b) access to services: promotion of access to education, healthcare and social assistance services through the fulfilment of conditionalities imposed by the programme; and c) intersectoral articulation: integration with other government actions such as job and income generation programmes, adult literacy and professional training. The BFP aims to break the long-term cycle of poverty for future generations by promoting access to healthcare and social services. This includes keeping child immunizations up to date, monitoring child growth and development, and maintaining high levels of school attendance. Pregnant women must undertake all planned prenatal visits.

Studies demonstrate the health impacts of the BFP – particularly for poverty related conditions. This includes reductions in under-5 mortality by diarrhoea and malnutrition, where the greater reductions have been in municipalities where BFP coverage of the target population has been 100% for at least 4 years [11]. The BFP is associated with reductions in the new case detection rate of leprosy, particularly in municipalities with high primary care coverage, and with greater leprosy multidrug therapy adherence and cure rates among multibacillary cases [12, 13]. Additionally, the BFP has been associated with reductions in incidence, treatment dropout, and deaths from tuberculosis and with an increase in the cure rate [14–17]. International evidence also supports the positive effects of CCTs on health outcomes, such as reductions in infant and maternal mortality. CCTs also affect the health status of adult recipients with lower incidences of chronic diseases such as diabetes, hypertension and obesity for CCT beneficiaries [18, 19].

It is plausible that BFP could reduce malaria incidence by alleviating poverty and improving access to healthcare. Evidence identifies how poverty enhances the risk of becoming infected [20]. In addition, an increase in the use of healthcare services, widely reported across international CCT programmes, can raise the chances of obtaining timely and appropriate diagnosis and treatment. This can reduce further transmission and consequently disease incidence [19]. Despite the potential for CCTs to affect malaria, this relationship has not been robustly explored [21].

This study investigated the association between the expansion of the BFP in Brazil and malaria incidence in endemic Brazilian municipalities between 2004 and 2015. It uses high-quality population-based data aggregated at the municipal-level and 12 years of observation and employs robust quasi-experimental methods. Furthermore, the analyses adjust for important contextual factors known to be important risk factors for malaria infection that could confound any relationship between the BFP and malaria incidence.

## Methods

### Study design and variables

A longitudinal ecological study (panel regression) was conducted using data from national administrative databases, which covered the period 2004 to 2015. The unit of analysis was the municipality. Descriptive analysis of trends of malaria incidence and for all the variables along the period under study was performed, after which regression models were fitted to verify the association between the BFP coverage and malaria incidence.

From 5560 Brazilian municipalities in 2003, 807 (out of 808) situated in the endemic states for malaria were selected. One municipality of the Pará state, created in 2013, was excluded due to limited data. The study area covers 5,084,130.557 km^2^ and, in 2019, comprised 28,990,627 inhabitants. This corresponds to approximately 60% of the national territory of Brazil, but only 14% of the national population.

The main outcome variable was the annual municipal incidence of malaria. It was calculated by dividing the total number of cases notified during the year by the population of the municipality. The main exposure was municipal BFP coverage. It was defined as the number of individuals enrolled in the BFP (obtained by multiplying the number of beneficiary families by the average family size) divided by the total population of the municipality. This variable also operates as a proxy for the penetration of the BFP into a municipality and captures increased financial resources and use of public services (health, education and social assistance) from the fulfilment of conditionalities. The annual population of municipalities and socioeconomic covariates were obtained from 2000 and 2010 demographic censuses and linear interpolation and extrapolation were employed for non-census years.

A total of 23 covariates, known to be determinants of malaria infection and that were available at the municipality level, were selected to include in the analyses. These included: population density (ratio between population and total area of the municipality, expressed as inhabitants per square meter); annual increase in deforestation (difference between deforested area in the current and the previous year presented in square meters, data were available only for 760 among selected municipalities); GINI index (measures the degree of income concentration through the difference between the income of the poorest and the richest, values varying between 0 and 1, where zero indicates that there is no inequality and 1 when the inequality is maximum); proportion of young adult men (percentage of male population aged between 20 and 49 years over the total population of the municipality, a group with a higher likelihood of contact with the mosquito); and the municipal human development index – MHDI (consists of the geometric mean of the indices of the 3 dimensions: income, education and longevity, with equal weights and a value varying between 0 and 1 – the closer to 1, the greater the human development level). Other covariates were: the proportion of indigenous population (percentage of the self-declared indigenous in the demographic census over the municipal population); hospital beds density (total hospital beds (public and private) per 1000 inhabitants), proportion of persons employed in the mineral extraction sector aged 18 years or over, gross domestic product at Real (BRL, the Brazilian currency) values – (GDP), proportion of beneficiaries of health insurance plans (percentage of the population that holds health insurance plans that contain hospital and/or outpatient segmentation, and may also contain dental assistance over the population), Family Health Programme (FHP) municipal coverage (percentage of the number of people registered in the FHP in December of each year over the municipal population), the ratio of average income of the richest 20% to the poorest 40%, the ratio between population living in rural and urban areas, and the unemployment rate among people aged 18 and over. Additionally, the following variables complete the list: educational dimension of the MHDI (geometric average of the subindex of children and young people’s school attendance, with a weight of 2/3, and the subindex of schooling of the adult population, with a weight of 1/3); longevity dimension of the MHDI (life expectancy at birth, through the formula: [(observed value of the indicator) - (minimum value)] / [(maximum value) - (minimum value)], where the minimum and maximum values are 25 and 85 years, respectively); income dimension of the MHDI (income per capita, through the formula: [ln (observed value of the indicator) - ln (minimum value)] / [ln (maximum value) - ln (minimum value)], where the minimum and maximum values are R$ 8.00 and R$ 4033.00 at August 2010 prices); public hospital beds density (proportion of the total number of existing hospital beds in the public sector per 1000 inhabitants); proportion of persons employed in the agricultural sector aged 18 or over (ratio between the number of persons aged 18 years and over employed in the agricultural sector and the total number of employed persons in this age group); proportion of extremely poor (percentage of extremely poor, defined as monthly income per capita up to R$ 70, 2010 prices, over municipal population); per capita income (ratio between the sum of the income, in Brazilian Reais, of all individuals living in permanent private households and the total number of these individuals, 2010 values corrected by the 2015 IP CA deflation factor); illiteracy rate among people aged 15 and over (percentage of the population aged 15 and over who cannot read or write a simple note over the total number of people in this age group); people living in households with bathroom and running water (percentage of the population living in permanent private households with running water in at least one of its rooms and with an exclusive bathroom over the total population living in permanent private households) (Additional files 3 and 4).

After conducting correlation analysis among the 23 selected covariates, nine were discarded (the last ones listed above), 14 were considered for model selection and eight were retained in the final model. The inclusion of covariates in the multivariate model followed the decreasing order of the incidence rate ratio of the bivariate analysis with the outcome. Non-significant variables were excluded from the model (*p* < 0.05). When the entry of a variable changed the *p*-value of a variable previously inserted in the model, the choice was made based on theory and the variable considered most relevant was retained.

### Data sources

Several information systems were consulted to obtain data for this study. From the Ministry of Health we used the Primary Health System for the FHP coverage (SIAB, *Sistema de Informação da Atenção Básica*) (http://www2.datasus.gov.br/SIAB/index.php?area=04), the National Register of Health Establishments (CNES, *Cadastro Nacional de Estabelecimentos de Saúde*) for the data on availability of hospitalization beds (http://www2.datasus.gov.br/DATASUS/index.php?area=0901), and the National Notifiable Disease Information System (SINAN, *Sistema de Informação de Agravos de Notificação*) (http://tabnet.datasus.gov.br/cgi/deftohtm.exe?sinannet/cnv/malabr.def) and Malaria Epidemiological Surveillance Notification System (SIVEP-Malária, *Sistema de Informação de Vigilância Epidemiológica da Malária*) (https://public.tableau.com/app/profile/mal.ria.brasil/viz/Dadosparacidado_201925_03_2020/Incio). For health insurance coverage information, data were obtained from systems managed by the National Health Agency (*Agência Nacional de Saúde)*, a regulatory agency linked to the Ministry of Health: Beneficiary Information System (*Sistema de Informação de Beneficiários*), Operator Registration System (*Sistema de Cadastro de Operadoras*) and the Product Registration System (*Sistema de Registro de Produtos*) (http://www.ans.gov.br/perfil-do-setor/dados-e-indicadores-do-setor/baixar-base-de-dados). For information on municipal BFP coverage, the Social Information Matrix (*Matriz de Informação Social*), maintained by the Ministry of Citizenship – former Ministry of Social Development and Fight Against Hunger –, was accessed (https://aplicacoes.mds.gov.br/sagi/portal/). Data on deforestation were collected at the PRODES Project that realizes the monitoring of the Amazon Forest by satellite, managed by the National Institute of Space Research (*Instituto Nacional de Pesquisas Espaciais*) of the Ministry of Science, Technology, Innovation and Communication (http://www.obt.inpe.br/OBT/assuntos/programas/amazonia/prodes). The MHDI was obtained at The Atlas of Human Development in Brazil Database (*Atlas do Desenvolvimento Humano no Brasil*) (http://www.atlasbrasil.org.br/) platform maintained by the United Nations Development Program Brazil, Institute of Applied Economic Research (*Instituto de Pesquisa Econômica Aplicada*), a federal public foundation linked to the Ministry of Economy, and the João Pinheiro Foundation, a research and teaching institution linked to the Secretariat of Planning and Management of the state of Minas Gerais, which adapted the methodology of the Global Human Development Index to calculate the MHDI of the Brazilian municipalities from the Brazilian Institute of Geography and Statistics (IBGE, *Instituto Brasileiro de Geografia e Estatística*) Demographic Censuses of 1991, 2000 and 2010. Socioeconomic and demographic variables were obtained from the 2000 and 2010 IBGE Demographic Censuses (https://sidra.ibge.gov.br/home/pmc/brasil) [2, 3, 22–25].

### Statistical analysis

Descriptive analyses were undertaken, including exploring the number of malaria cases, the API, BFP municipal coverage and all covariates over the period of study. Multivariable conditional negative binomial regression modelling was used for panel data with fixed effects. Panel regression modelling was chosen as the most appropriate method to model hierarchical data (i.e. yearly observations clustered by municipalities) and capture changes over time. Fixed effects specifications were chosen to adjust for municipal-level fixed effects and to control for unobserved time-invariant characteristics of municipalities. These variables would be geographical, historical, or socio-cultural characteristics of each municipality. Fixed effects panel regression is a robust and well-employed method in health systems research and policy evaluation [26]. A negative binomial specification was chosen as appropriate for modelling count data (e.g. cases) which are overdispersed (i.e. the mean is not equal to the variance). The models were specified with malaria cases (counts) as the outcome and included an offset term (population) therefore modelling rates. The effect estimates were expressed as incidence rate ratios (IRR) and interpreted as the relative ratio in malaria incidence rates given a one unit change in explanatory variables. Stata version 12.0 was used.

## Results

The annual total cases of malaria in the 807 selected municipalities decreased by 70% between 2004 and 2015 from 454,353 to 138,109, respectively. The total of these autochthonous new cases of malaria represented 99.9% of total cases in Brazil over the period [3]. Out of the 807 municipalities, 81 (10%), located in the state of Tocantins (55), Maranhão [17] and Mato Grosso [9], did not register any autochthonous cases during the study period. Autochthonous cases in Brazil result from infections by *P. vivax*, *P. falciparum* and *P. malariae*, and in 2015, 89% of the cases were caused by *P. vivax*. The percentage contribution of cases of *P. falciparum* malaria, known to be more severe and lethal, or mixed malaria – infections with more than one species of *Plasmodium* – dropped from 23% in 2004 to 11% in 2015. Cases became more geographically concentrated. In 2004, autochthonous malaria cases occurred in 622 municipalities, while in 2015 only 298 municipalities registered new autochthonous cases. Regarding the species, cases due to *P. vivax* infection in 2004 were recorded in 591 municipalities whilst and *P. falciparum* malaria was reported in 460 municipalities. In 2015 the reported cases by species fell to 295 and 129 municipalities, respectively.

The number of high-risk municipalities (API equal to or higher than 50 cases per thousand inhabitants) decreased from 91 in 2004 to 29 in 2015. The overall API decreased from 19.6 to 5 cases per thousand inhabitants in 2015. The API from *P. falciparum* and mixed malaria ranged from 4.58 to 0.56 cases per thousand inhabitants for the same period (Additional file 1).

Table 1 shows bivariate analysis between malaria API and the 14 covariates used for model selection. The following variables were retained in the final model: gross domestic product, proportion of young adult men, proportion of beneficiaries of health insurance plans, population density, annual increase in deforestation, FHP municipal coverage, unemployment rate among people aged 18 and over and GINI index.

**Table 1:** Fixed-effect negative binomial models for association between malaria incidence and Bolsa Família coverage, Brazil 2004–2015

	Malaria Incidence Risk Ratio (95% CI)	
	Covariates used for model selection	
	Crude	Adjusted
Municipal human development index	0.000 (0.0001–0.0002)	–
Hospital beds density	0.958 (0.9292–0.9888)	–
Ratio of average income of the richest 20% to the poorest 40%	1.000 (1.0004–1.0010)	–
Ratio between population living in rural and urban areas	1.147 (1.1329–1.1612)	–
Proportion of indigenous population	1.034 (1.0318–1.0375)	–
Proportion of persons employed in the mineral extraction sector aged 18 years or over	1.03 (1.0141–1.0458)	–
	**BFP models**	
BFP municipal coverage (%, 0–100)	**0.987 (0.9852–0.9885)**	**0.997 (0.9948–0.9988)**
Logarithm of gross domestic product (BRZ, prices 2015)^a^	0.479 (0.4653–0.495)	0.628 (0.6019–0.6556)
Proportion of young adult men^b^	0.892 (0.8830–0.902)	1.057 (1.0476–1.0673)
Proportion of beneficiaries of health insurance plans (%, 0–100)	0.903 (0.8957–0.9098)	1.01 (1.0026–1.0175)
Population density (number of people/km ^2^)	0.997 (0.9966–0.9973)	0.9989 (0.9986–0.9992)
Annual increase in deforestation (Km^2^)	1.002 (1.0022–1.0026)	1.000 (1.0001–1.0007)
FHP municipal coverage (%, 0–100)	1.012 (1.011–1.0141)	0.9976 (0.9961–0.9992)
Unemployment rate among people aged 18 and over (%)	1.02 (1.0318–1.0482)	0.9755 (0.9661–0.9851)
GINI index (0–100)	1.048 (1.0426–1.0531)	1.0280 (1.0220–1.0340)
Year	–	0.85 (0.8409–0.8595)
Number of observations	8544	7729
Number of municipalities	712	670

^a^The Log of gross domestic product was used in the model building strategy

^b^ Adults between 20 and 49 years old

**Fig. 1:**
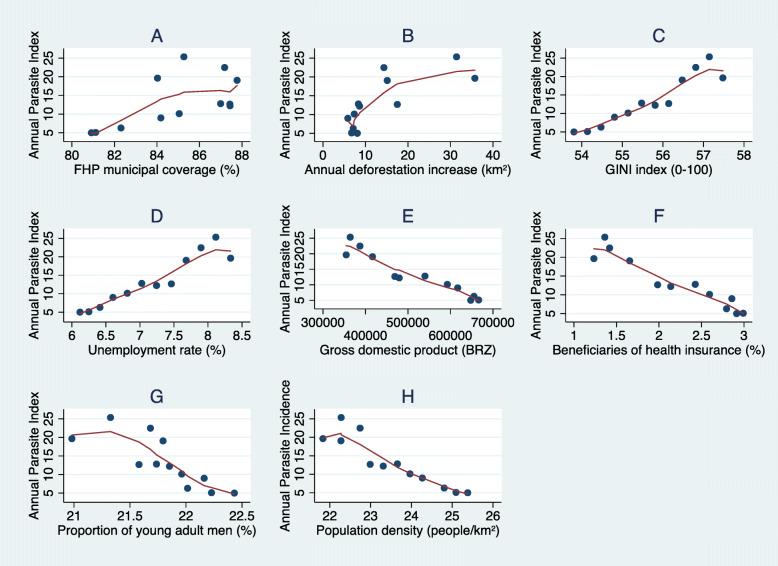
Distribution of the annual malaria index with covariates retained in the final model. Locally Weighted Scatterplot Smoothing of the annual malaria incidence with the annual mean values of the eight covariates retained in the selected model, over the period from 2004 to 2015: **A** Family Health Programme (FPH) municipal coverture in percentage; **B** Annual increase in deforestation measured in square kilometres; **C** GINI index ranging from 0 to 100; **D** Unemployment rate among people 18 years old and over; **E** Gross domestic product in Brazilian Reais (2015 prices); **F** Proportion of beneficiaries of health insurance plans over the municipal population; **G** Proportion of men aged between 20 and 49 years old; and **H** Population density expressed in terms of inhabitants per square kilometres

The mean BFP municipal coverage increased over the study period, from 21.71% in 2004 to 45.71% in 2015. The mean FHP coverage decreased from 84.02 to 80.91%. Furthermore, there was a decrease in the municipal deforestation rate (from 35.74 km^2^ to 8.08 km^2^), in the mean GINI index (from 57.48 to 53.81), and in the unemployment rate among people aged 18 and over (from 8.33 to 6.12%). The average gross domestic product total of municipalities increased in 82.6% in the selected municipalities during the period (R$354,734 to R$647,722). Moreover, the average population with private health insurance increased from 1.23% in 2004 to 2.91% in 2015. The proportion of male population aged between 20 and 49 years was 22.43% in 2015, compared to 20.98% in 2004. At the same time, there was an increase of 16.26% in population density in the Amazonian municipalities (Fig. 1, Additional file 2).

Table 1 shows crude and adjusted association (from adjusted fixed effects negative binomial regression models) between malaria incidence with BFP coverage. An increase in the BFP municipal coverage was associated with a significant reduction in malaria incidence before and after controlling for demographic, socioeconomic and time-dependent variables. In the adjusted model, one percentage point increase in the BFP municipal coverage was associated with a 0.3% decrease (IRR: 0.997, 95%, CI: 0.995–0.999) in malaria incidence. Programmatically, it can be assumed that increasing the BFP municipal coverage in 10% would lead to 3% decrease in the malaria incidence. Given the average municipal BFP coverage increased by 24 percentage points over the period 2004–2015, this corresponds to be a reduction of 7.2% in the malaria incidence associated with BFP expansion.

## Discussion

This study identified that increases in BFP coverage were associated with reductions in the incidence of malaria in 807 municipalities of the Brazilian Amazon. Furthermore, this effect is adjusted for a wide range of socioeconomic and health system factors that could confound the relationship.

A plausible explanation for these findings is that increasing BFP coverage increases access to existing public health services. This is in line with evidence from other CCT experiences that reflect in lower incidences [19]. By meeting health conditionalities, families access health services more frequently, increasing the chances of detecting new cases and contributing to the interruption of malaria transmission, resulting in the reduction of disease incidence. The negative association between the FHP coverage and the malaria incidence reinforces this hypothesis and suggests that greater coverage of health services could facilitate access to diagnosis and treatment, thus improving its control.

Additionally, the BFP may reduce malaria incidence by improving household living standards and a reduced risk of contracting malaria. This would be expected from improvements in housing quality, such as roof, walls, or insect screens which can reduce the exposure to mosquitoes. However, despite evidence of decreased poverty and improved living conditions among CCT recipients, there is little evidence on substantial impact on the physical structure of households [18, 20]. Beneficiaries of the BFP may also take up preventive measures more often or actively seek out early treatment due to better socioeconomic status and reduced burden from poverty [20]. However, diagnosis, treatment and other malaria control measures are available free of charge in Brazil and geographical barriers are important for access in the Amazon context, so this pathway may be less relevant. Lastly, better nutritional and health status of CCT beneficiaries could improve malaria outcomes resulting in reduced chances of onward transmission [18].

Transmission of malaria can certainly be reduced with investments in personal expenses in preventive methods, increased funding for government programmes to control the disease and development in general such as urbanization. However, when compared to other important endemic diseases to poor countries, such as diarrhoea, tuberculosis and schistosomiasis that are influenced by precarious living conditions, the fact that malaria is a vector-borne disease makes it strongly influenced by environmental conditions [6–10, 27, 28]. This feature also makes the causal chain linking poverty to the risk of becoming ill more complex. According to the malaria frontier concept, which describes the dynamics of malaria transmission in new settlements in the Amazon region, the first years after human arrival show high rates of malaria transmission. At this stage, risks mainly derive from environmental transformations, which promote larval habitats for *Anopheles darlingi*, the most important malaria vector in the Brazilian Amazon. Decreasing infection rates 10 years after the establishment of settlements would be associated in part with improvements in the quality of life resulting from economic gains such as agriculture, ranching and urban development [29, 30]. Interaction between environmental and socioeconomic factors would determine the pattern of disease distribution in land transformation scenarios [31]. The positive association found between the API of malaria and the annual deforestation increase and with GINI index would corroborate this observation. Also, the negative correlation between malaria incidence with GDP and with population density would also support this reasoning.

The present study has some limitations. First, there was interpolation and extrapolation of the 2000 and 2010 census variables, which may have introduced bias. We have assumed trends in these variables were linear. Second, there may be potential confounders that we could not adjust for. Information was not available on vector control measures such as the distribution and installation of long-lasting insecticide-treated mosquito nets, residual indoor spraying or environmental management actions. Thirdly, the ecological approach precludes individual-level inference. The analysis of ecological data implies the risk of considering relationships observed at the aggregate level to be valid at the individual level, which is known as the ecological fallacy. Nevertheless, the unavailability of all data at individual level and the relevance of the question justify the effort to produce and discuss the findings.

However, there are key strengths to this study. The quality of data for a middle-income country is high and facilitates robust research. In Brazil, cases of malaria are defined based on laboratory diagnosis performed free of charge in the Unified Health System (*Sistema Único de Saúde*) facilities. This likely reduces the possibilities of under-reporting and provides consistent comparable trends. In addition, 12 years of observation of population-based data were employed for all the endemic municipalities in the country producing a large and long panel dataset.

The findings of this study build on the wealth of evidence demonstrating the health-improving benefits of CCTs in low and middle-income countries. Despite the complexities of malaria transmission and its relationship with poverty, this study identifies how poverty-alleviation policies coupled with incentives for healthcare use can reduce malaria incidence. Policy-makers should consider the wider health-benefits of CCTs in the context of free and universal health care. Brazil has a strong primary healthcare system and free malaria treatments, which may be essential for the effectiveness of CCT programmes to reduce malaria incidence.

## Conclusion

The present study identifies a reduction in the incidence of malaria associated with increased coverage of a large CCT programme in Brazil. In addition to reducing poverty, there is likely greater access to health services as a consequence of complying with conditionalities, which may contribute to decreasing malaria incidence. CCT programmes, coupled with strong public healthcare, are valuable for reducing poverty and infectious disease burdens, and maximising these synergies should be a priority for policymakers.

## Supplementary Information

**Additional file 1. **Annual parasite index (API): total and by species – *P. vivax*, *P. falciparum* and mixed infections and *P. malariae* – between 2004 and 2015*.* Tabular data provided as an Excel spreadsheet (XLS) containing the annual parasite index (API): total and by species – *P. vivax*, *P. falciparum* and mixed infections and *P. malariae.*

**Additional file 2.** Characteristics of 807 selected municipalities between 2004 and 2015. Tabular data provided as an Excel spreadsheet (XLS) containing information on the behaviour of the annual average values of the covariates over the period between 2004 and 2015 for the BFP, Bolsa Família Programme; FHP, Family Health Programme. The standard deviation is shown in brackets.

**Additional file 3.** Database analysed. Tabular data containing database analysed presented in a CSV file.

**Additional file 4.** Dictionary of variables. Dictionary of variables in the analysed database.

## Data Availability

All data analysed during this study are included in Additional file 3. Public access to the databases is open and can be obtained through the following links: Primary Health System (SIAB, *Sistema de Informação da Atenção Básica*): http://www2.datasus.gov.br/SIAB/index.php?area=04; National Register of Health Establishments (CNES, *Cadastro Nacional de Estabelecimentos de Saúde*): http://www2.datasus.gov.br/DATASUS/index.php?area=0901; National Notifiable Disease Information System (SINAN, *Sistema de Informação de Agravos de Notificação*): http://tabnet.datasus.gov.br/cgi/deftohtm.exe?sinannet/cnv/malabr.def; Malaria Epidemiological Surveillance Notification System (SIVEP-Malária, *Sistema de Informação de Vigilância Epidemiológica da Malária*): https://public.tableau.com/app/profile/mal.ria.brasil/viz/Dadosparacidado_201925_03_2020/Incio; Beneficiary Information System (*Sistema de Informação de Beneficiários*), Operator Registration System (*Sistema de Cadastro de Operadoras*) and the Product Registration System (*Sistema de Registro de Produtos*) from the National Health Agency (*Agência Nacional de Saúde)*: http://www.ans.gov.br/perfil-do-setor/dados-e-indicadores-do-setor/baixar-base-de-dados; Social Information Matrix (*Matriz de Informação Social*): https://aplicacoes.mds.gov.br/sagi/portal/; PRODES Project: http://www.obt.inpe.br/OBT/assuntos/programas/amazonia/prodes; Atlas of Human Development in Brazil Database (*Atlas do Desenvolvimento Humano no Brasil*): http://www.atlasbrasil.org.br/; and IBGE Brazilian Institute of Geography and Statistics (IBGE, *Instituto Brasileiro de Geografia e Estatística*): https://sidra.ibge.gov.br/home/pmc/brasil. For this research data were obtained from the links mentioned above, except for the SINAN and SIVEP-Malária data, which were requested directly from the Ministry of Health of Brazil.

## Abbreviations

API: Annual parasite index
BFP: Bolsa Família Programme
CCT: Conditional cash transfer
FHP: Family Health Programme
GDP: Gross domestic product
IBGE: Brazilian Institute of Geography and Statistics
IPCA: Broad national consumer price index
IRR: Incidence rate ratio
MDHI: Municipal human development index
